# No Sweat! Bilateral Shoulder Reduction Using a Modified Davos Technique

**DOI:** 10.5811/cpcem.2018.11.39445

**Published:** 2019-01-22

**Authors:** Jijoe Joseph, Nancy Nguyen, Daniel Gruzman, Anthony Boutin, Dean Olsen

**Affiliations:** Nassau University Medical Center, Department of Emergency Medicine, East Meadow, New York

## Abstract

Shoulder dislocations are a common entity seen and treated in the everyday practice of emergency physicians. Bilateral simultaneous shoulder dislocations, however, are rare and are only described in the literature through case reports with no consensus about how to effectively and efficiently reduce them. We present a case of a 21-year-old male who sustained bilateral simultaneous anterior shoulder dislocations after a suspected seizure. Following confirmation with radiographs, the patient’s dislocations were reduced successfully and in a timely manner using a novel method: the modified Davos technique.

## INTRODUCTION

Anterior shoulder dislocation is a well-known injury encountered in the emergency department (ED). Bilateral shoulder dislocations, however, are rare and may present as both posterior and anterior types. Of the two, bilateral posterior shoulder dislocations are far more prevalent than bilateral anterior shoulder dislocations.^1^ Bilateral posterior dislocations are traditionally caused by sports injuries, seizures, electrical shock or electroconvulsive therapy. Simultaneous bilateral anterior shoulder dislocations on the other hand are usually of traumatic origin with only a few cases described in the literature.^1^ After a thorough literature search, we found select cases that reported simultaneous bilateral anterior shoulder dislocations following seizures.^5^–^7^

## CASE REPORT

A 21-year-old incarcerated male was brought by ambulance to the ED for evaluation of bilateral shoulder pain. Prior to arrival the patient had suffered a witnessed, generalized tonic-clonic seizure at the penitentiary. He was stabilized by on-site medical personnel at the institution. During examination in the ED, the patient was lucid and oriented despite transitioning out from a postictal state. Upon questioning, he reported shoulder pain that he described as similar bilaterally – moderate to severe in intensity, sharp in nature with generalized radiation to adjacent joint structures and apprehensive to movement secondary to discomfort. He denied numbness, tingling and presence of stingers of the affected upper extremities. On physical exam, the patient was in mild distress, both shoulders resting in slight abduction and external rotation.

Both humeral heads were palpated along the anterior aspect of each glenohumeral joint with global, painful restriction of range of motion bilaterally without any evidence of peripheral motor, sensory or vascular deficit.

**Image 1:**
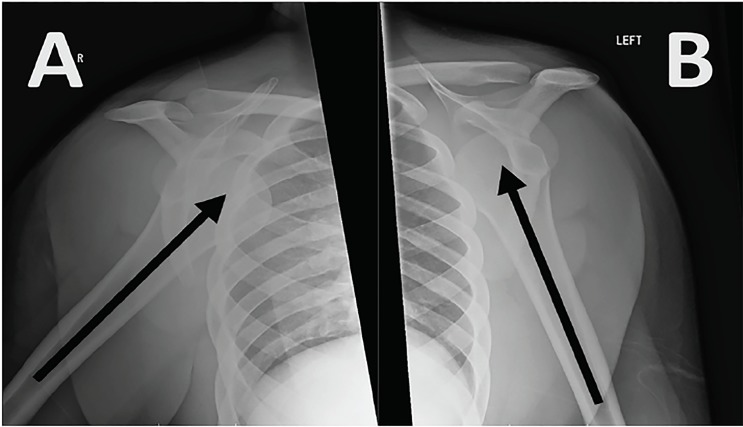
Findings on arrival. (A) Shoulder radiograph showing anterior dislocation of the right shoulder joint (arrow). (B) Shoulder radiograph showing anterior dislocation of the left shoulder joint (arrow).

**Image 2:**
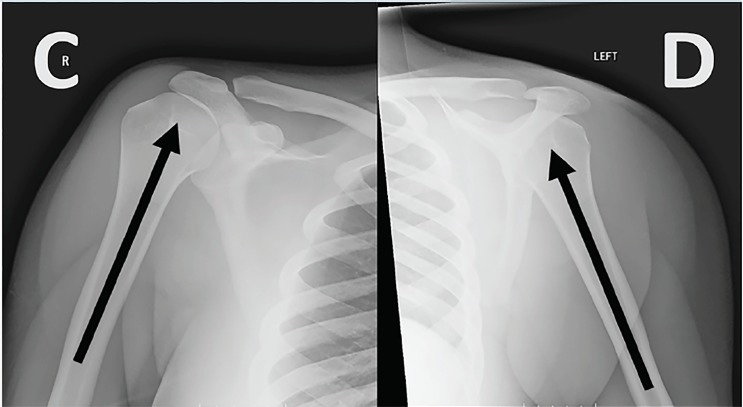
Post-reduction films of the right (C) and left (D) shoulders following the modified Davos procedure. There is anatomic alignment of both glenohumeral joints, and no fractures are identified.

Plain radiographs confirmed bilateral subcoracoid dislocations, with the humeral heads lying anteriorly, medially and inferiorly in respect to the glenoid fossae (Image 1). We performed prompt reduction using modified Davos technique without anesthesia or analgesia, followed by sling immobilization and subsequent rehabilitation (Image 2).

## DISCUSSION

Simultaneous bilateral glenohumeral joint dislocations, with or without fractures, in all planes are rare. The force necessary to produce a dislocation must act in synchrony in both joints.^2^ Bilateral shoulder dislocations were first described in 1902 in a patient with excessive muscle contractions that occurred as a result of camphor overdose.^2^ Evidence from the literature suggests that most bilateral dislocations are posterior. 4–5 Bilateral anterior dislocations are rare. In 1999, Dinopoulos et al. found only 28 reported cases since 1966.^3^

Numerous techniques are employed by emergency physicians to reduce anterior shoulder dislocations. Most involve traction and/or rotation of the glenohumeral joint in some capacity that may result in excessive pain, sometimes requiring procedural sedation. In response to this, Boss, Holzach and Matter in 1993, working at Davos hospital in Switzerland, introduced an alternative that was later coined the Davos technique, a non-traumatic, patient-controlled and auto-reduction method that would obviate the need for pain medications or anesthesia and the associated risks. Boss et al. demonstrated a 60% success rate with the Davos technique.^8^–^9^

Over the past three decades, this technique has been gaining popularity. Reports were published by Ceroni et al. in 1997, Stafylakis et al. in 2016, and Marcano-Fernández et al. in 2018. This technique has consistently been found to have an improving success rate with the most recent being 77%.^10^–^12^ This is comparable to other reduction methods (notably the Milch technique, 82–89%).^11^ The only requirements are a conscious patient, an elastic bandage, and an assistant. The Davos technique can be performed safely by trained personnel; this technique decreases the time to reduction in addition to reducing the patients’ pain and anxiety levels. It can be particularly useful to those patients with a risk of recurrence or in remote locations with no immediate access to a hospital.^12^ A caveat to the implementation of this technique is that it requires a high degree of communication between practitioner and patient as well as compliance from the patient.^11^ Below are the steps to perform the Davos technique as originally described by Boss et al.

**CPC-EM Capsule**

What do we already know about this clinical entity?

*Bilateral anterior shoulder dislocations are rare and usually the result of trauma. The Davos technique is a patient-controlled method of anterior shoulder dislocation reduction*.

What makes this presentation of disease reportable?

*This was the first case found in literature in which a simultaneous bilateral anterior shoulder dislocation was reduced using a modified version of the Davos technique*.

What is the major learning point?

*The Davos technique can be modified to treat bilateral simultaneous anterior shoulder dislocations successfully without the risks of procedural sedation*.

How might this improve emergency medicine practice?

*This case, despite being rare, represents a safe and cost-effective way to treat an entity with no clear consensus regarding management which could prove useful to clinicians who encounter it*.

### The Standard Davos Reduction Procedure

Proper positioning for the procedure includes the patient sitting on the bed holding the injured extremity with their non-injured hand with their ipsilateral knee flexed as much as possible. The physician then ties both hands together using an elastic band or tape sparing the fingers with elbows kept close to thigh (patient should avoid crossing fingers as this can lead to an increased muscle tension). The physician or an assistant sits on the patient’s foot to help stabilize the wrist against the anterior tibia and instructs the patient to lean his head back and let his shoulders roll forward (shrug) as he slowly tries to lie back in bed. This neck extension exerts traction on the injured shoulder. A successful reduction should be followed by standard post-reduction intervention.^8^–^9^

As our patient had bilateral anterior shoulder dislocations, we modified our approach to this technique to adapt to his situation, which we describe below. To the best of our knowledge, this is the first reported use of such a technique to treat bilateral anterior shoulder dislocations caused by a seizure.

### Modified Davos Procedure

1. The patient was instructed to sit with his back resting on the back of the stretcher and the bed at 90–100 degrees. Each of the patient’s arms was individually tied to a sheet.
2. One physician held gentle traction to the sheet so that the patient’s arms were lifted parallel to the ground while another physician slowly lowered the head of the bed, asking the patient to extend his head backward and keep his back resting against the stretcher.
3. Dislocation is then promptly reduced, and standard post-reduction intervention should follow.

## CONCLUSION

In summary, because bilateral anterior shoulder dislocations are rare there is uncertainty regarding their management. Employing the modified Davos-reduction procedure described in this case report to treat this entity yielded multiple benefits: the patient was able to control his reduction by using his own weight; no sedation or analgesia was necessary; and the provider performing the procedure did not need to exert any physical effort other than to hold traction on a sheet to keep the patient’s arms elevated. Although limited in its scope of use and requiring further study, we are optimistic about the utility of this novel procedure to treat bilateral anterior shoulder dislocations as it exposes the patient to virtually no risk and potentially major benefit.

## References

[1] Page AE, Meinhard BP, Schulz E (1995). Bilateral posterior fracture-dislocation of the shoulders: Management by bilateral shoulder hemiarthroplasties. J Orthop Trauma.

[2] Dinopoulos HT, Giannoudis PV, Smith RM (1999). Bilateral anterior shoulder fracture-dislocation. Int Orthop.

[3] Dunlop CC (2002). Bilateral anterior shoulder dislocation: a case report and review of the literature. Acta Orthop Belg.

[4] Tripathy SK, Sen RK, Aggarwal S (2011). Simultaneous bilateral anterior shoulder dislocation: report of two cases and review of the literature. Chin J Traumatol.

[5] Auerbach B, Bitterman A, Mathew C (2015). Bilateral shoulder dislocation presenting as a unilateral shoulder dislocation: case report. J Am Osteopath Assoc.

[6] Lasanianos NG, Mouzopoulos G (2008). An undiagnosed bilateral anterior shoulder dislocation after a seizure: a case report. Cases J.

[7] Marty B, Simmen HP, Käch K (1994). Bilateral anterior shoulder dislocation fracture after an epileptic seizure. A case report. Unfallchirurg.

[8] Boss A, Holzach P, Matter P (1993). Analgesic-free self-reduction of acute shoulder dislocation. Z Unfallchir Versicherungsmed.

[9] Boss A, Holzach P, Matter P (1993). A new self-repositioning technique for fresh, anterior-lower shoulder dislocation. Helv Chir Acta.

[10] Ceroni D, Sadri H, Leuenberger A (1997). Anteroinferior shoulder dislocation: an auto-reduction method without analgesia. J Orthop Trauma.

[11] Stafylakis D, Abrassart S, Hoffmeyer P (2016). Reducing a shoulder dislocation without sweating. The Davos technique and its results. Evaluation of a nontraumatic, safe, and simple technique for reducing anterior shoulder dislocations. J Emerg Med.

[12] Marcano-Fernández FA, Balaguer-Castro M, Fillat-Gomà F (2018). Teaching patients how to reduce a shoulder dislocation: a randomized clinical trial comparing the Boss-Holzach-Matter self-assisted technique and the Spaso method. J Bone Joint Surg Am.

